# The role of lateralisation and sex on insular cortex: 3D volumetric analysis

**DOI:** 10.3906/sag-2010-137

**Published:** 2021-06-28

**Authors:** Fatma ÖZ, Niyazi ACER, Nihan KATAYIFÇI, Güneş AYTAÇ, Kamil KARAALİ, Muzaffer SİNDEL

**Affiliations:** 1 Department of Anatomy, Faculty of Medicine, Hatay Mustafa Kemal University, Hatay Turkey; 2 Department of Anatomy, Faculty of Medicine, Arel University, İstanbul Turkey; 3 Department of Physical Therapy and Rehabilitation, Faculty of Health Sciences, Hatay Mustafa Kemal University, Hatay Turkey; 4 Department of Anatomy, Faculty of Medicine, TOBB University of Economics & Technology, Ankara Turkey; 5 Department of Radiology, Faculty of Medicine, Akdeniz University, Antalya Turkey

**Keywords:** Insula of reil, MRI, brain mapping, sex differences

## Abstract

**Background/aim:**

The insula has attracted the attention of many neuroimaging studies because of its key role between brain structures. However, the number of studies investigating the effect of sex and laterality on insular volume is insufficient. The aim of this study was to investigate the differences in insular volume between sexes and hemispheres.

**Materials and methods:**

A total of 47 healthy participants [24 males (20.08 ± 1.44 years) and 23 females (19.57 ± 0.90 years)] underwent magnetic resonance imaging (MRI). Imaging was performed using the 3T MRI scanner. The insular volume was measured using the Individual Brain Atlases using Statistical Parametric Mapping (IBASPM); total intracranial, cerebral, grey and white matter volumes were measured using volBrain.

**Results:**

The right insular volume was significantly higher than the left insular volume in the participants, and the left cerebral volume was significantly higher than the right cerebral volume (p < 0.05). The total brain, total cerebral, left and right insular, and cerebral volumes were significantly larger in males than in females (p *<* 0.001). Also, the ratios of the insular volume to total brain and cerebral volume were significantly higher in males than in females (p *<* 0.05).

**Conclusion:**

This study shows that insular volume differs with laterality and sex. This outcome may be explained by the anatomical relationship between the insula and behavioural functions and emotional reactions and the fact that the right side of the brain is best at expressive and creative tasks.

## 1. Introduction

When you eat a chocolate, find the love of your life, smell a magical Bougainvillea, speak with your heart, or sadden with the tears of a young child, a hidden island is working hard: the insula (Island of Reil). The insular cortex is located on the lateral wall of the cerebral hemispheres and is fully covered by the parietal, frontal, and temporal opercula in the depths of the Sylvian fissure [1,2]. The insula is important in the processing of visceral sensory/motor, emotional, vestibular, pain, temperature and language inputs, in addition to visual, auditory, tactile, olfactory, and gustatory information [1–5]. 

The central sulcus of the insula is anatomically separated into two main sections, the anterior and posterior lobules, based on different cytoarchitectonics (granular, dysgranular and agranular), connectivity and functions. The anterior lobule includes three short gyri (anterior, middle and posterior), the posterior lobule has two long gyri (anterior and posterior), and the insula consists of the ventroanterior, dorsoanterior,, and posterior subregions [3,4]. The ventroanterior part is related to socioemotional processing (mainly receiving afferents from the limbic, entorhinal, perirhinal and posterior orbitofrontal cortices, and the cingulate gyrus), and the dorsoanterior part is linked to cognitive processing. The posterior insula is involved in auditory processing and somatovisceral sensations (receiving a projection from the viscera, which is relayed in the solitary tract nucleus, parabrachial complex, and thalamus) [1,6,7]. The insula displays activation during the processing of pain, but there is no consensus on the localisation of the pain-related activity [7]. Pain in the body, such as burns, activates the posterior insula, while feeling empathy for another person’s pain activates the anterior insula [4]. Moreover, the left and right insula have different functions [8]. Recent studies showed that the right insula was associated with the affective-perceptual form of empathy, olfaction, autonomic control of cardiac activity, self-awareness of actions, pain perception, singing, and the sympathetic system, while the left insula was associated with speaking, the parasympathetic system, and cognitive-evaluative forms of empathy [7,9–13]. 

Several studies on sex-based volume differences in specific parts of the brain have been reported in neuroimaging studies [14–17]. A meta-analysis investigating sex differences in the human brain showed that average total brain volume is higher in males than in females. In addition, volume and tissue density of the amygdala, hippocampus and insula differ by sex. Regional sex differences are found in a number of areas, including those known to be implicated in sex-based neuropsychiatric conditions [18]. 

The number of studies investigating differences in insular volume based on sex and laterality is insufficient. Thus, we aimed to investigate these differences between sexes and hemispheres.

## 2. Materials and methods 

### 2.1. Subjects 

In this retrospective study, we analysed magnetic resonance images (MRI) from a total of 47 healthy participants (23 females and 24 males) who were scanned between February 2015 and December 2016 in the department of Radiology at Akdeniz University. The study was approved by the local ethics committee (Akdeniz University, protocol no.: 2015.02.25) and written informed consent was obtained from each subject, in accordance with the Declaration of Helsinki. Participants were students from the faculty of medicine. The females and males had similar ages (M: 20.08 ± 1.44 years, range 18–25 years, F: 19.57 ± 0.90 years, range 18–22 years). Eleven males and 9 females were right-handed, and the rest were left-handed, as determined by the Edinburgh Handedness Inventory (Revised) [19]. Participants were excluded if they had any history of neurological, psychiatric or systemic disease, and all of them were free of any medications at the time of testing.

### 2.2. MRI acquisition

Imaging was performed using the 3T (Siemens, Spectra, Erlangen, Germany) MRI scanner. MRI protocol: 3D T1-MPRAGE TR (Repetition time)/TE (Echo time): 1900/2.41 ms; flip angle: 9’; Matrix: 256 × 186; FOV: 250 mm2; acquisition time: 3 min 21 s; number of axial slices: 176; slice thickness: 1 mm.

#### 2.2.1. Volume measurements

**Intracranial cavity volume**

**Figure 1:**
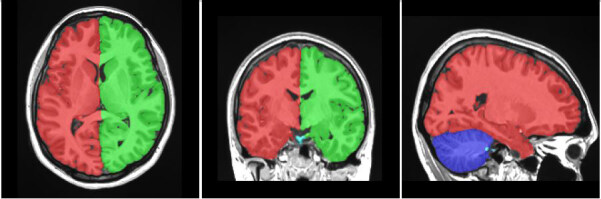
Segmentation of macrostructures of brain with volBrain.

To calculate the volumes of intracranial structures, such as total intracranial, cerebral, and grey and white matter volumes, processing was performed using volBrain (v.1.0, http://volbr ain.upv.es), a free online MRI brain volumetry system [20]. volBrain uses a fully-automated segmentation technique in which the algorithm is based on multi-atlas patch-based label fusion segmentation technology [20,21]. (Figure 1).

**Automated insular volumetry using IBASPM**

**Figure 2:**
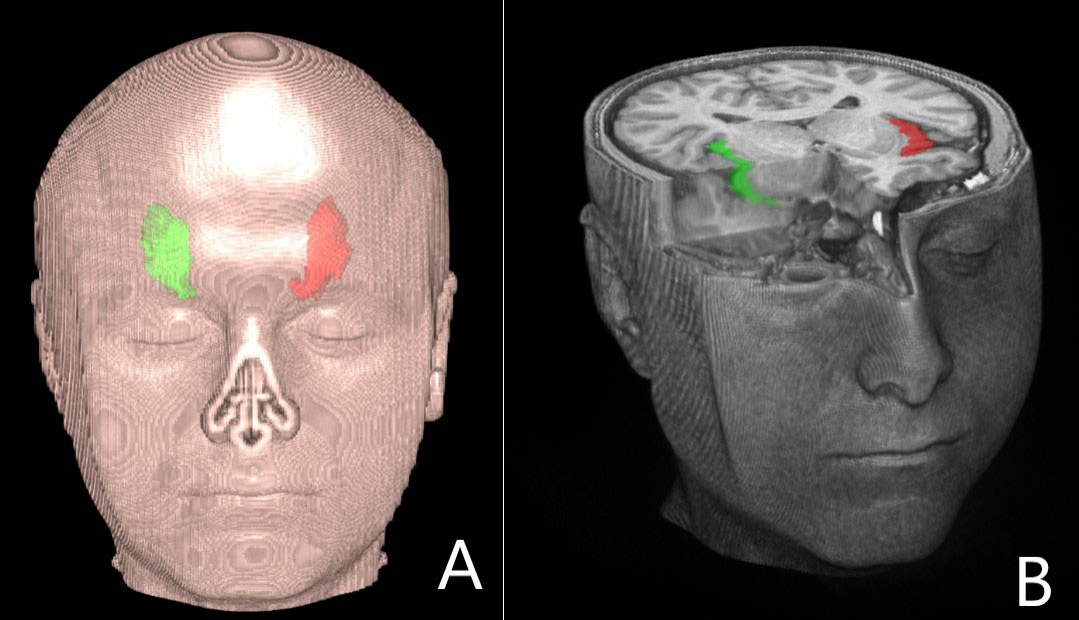
Insular cortex rendering on a T1 image: (A) coronal view; and (B) axial view showing the insula (red: left; green: right).

**Figure 3:**
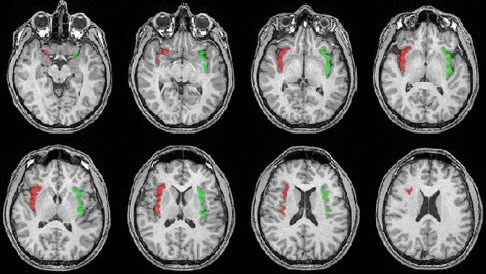
Insular cortex displayed on a T1 image: axial section showing the insula (red: left; green: right).

**Figure 4:**
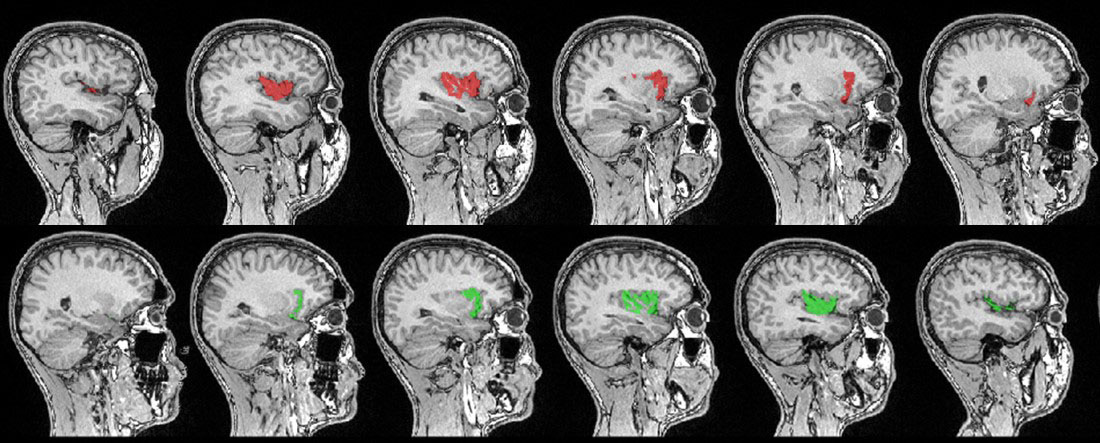
Insular cortex displayed on a T1 image: sagittal section showing the insula (red: left; green: right).

**Figure 5:**
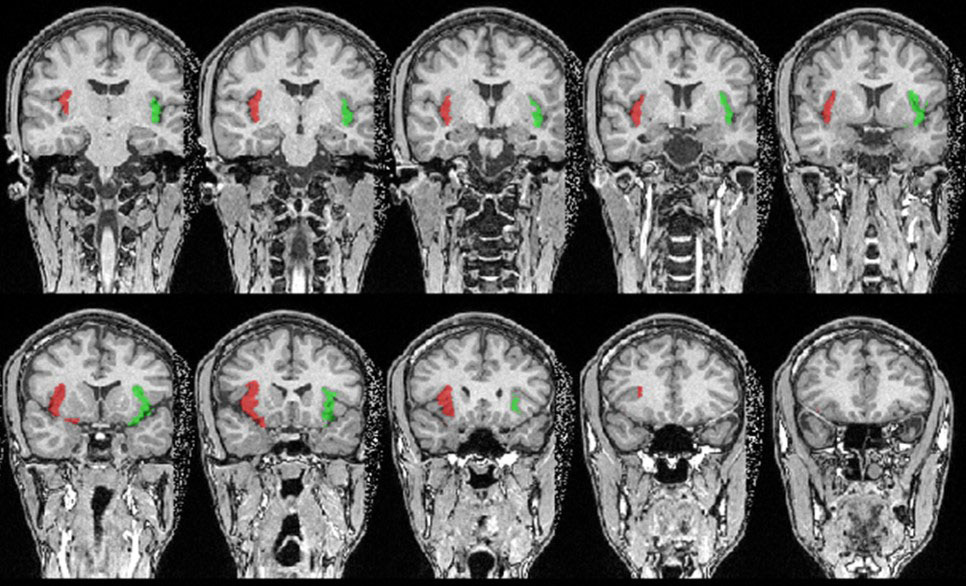
Insular cortex displayed on a T1 image: a coronal section showing the insula (red: left; green: right).

Automated segmentation of the insular volume was performed using IBASPM (Individual Brain Atlases using Statistical Parametric Mapping). We used MRIcron to convert scans into the NIfTI format (nii) to ensure compatibility with IBASPM. We used SPM8, implemented in MATLAB 10a (MathWorks, Natick, MA, USA), and the Automated Anatomical Labelling (AAL) atlas with 116 pre-defined segmentations. This process has four steps: segmentation, normalisation, labelling, and atlasing [1,22] (Figures 2A and 2B, Figures 3–5).

We segmented volumetric MRI images into grey matter, white matter and cerebrospinal fluid in native space, normalised to the ICBM 152 T1 template Montreal Neurological Institute (MNI) space to obtain the spatial transformation matrix. We automatically labelled each normalised individual grey matter voxel using the AAL atlas created from each subject’s MRI image atlased for the individual, and finally, the volumes of the generated atlases were automatically evaluated for 116 brain regions [23,24]. 

### 2.3. Statistical analysis

Statistical analyses were performed using the SPSS v:20.0 statistical package (IBM Corp., NY, USA). The normality of the data was tested using the Shapiro–Wilk test. The independent samples test was used to compare demographic and clinical characteristics of the male and female groups and differences between the groups were reported as mean ± standard deviation (mean ± SD), mean difference, and confidence interval (95% CI). A paired Student’s t-test was used to compare the left and right cerebrum measurements from the same subjects. For nondistributed data which were expressed as median (minimum–maximum), the Mann–Whitney U test was used to compare the sexes, and the Wilcoxon test was used to compare the left and right sides of each subject’s brain [25]. A p-value ≤ 0.05 was considered to be statistically significant. The required sample size for the study was calculated using the G*Power software (G*Power, Version 3.1.9.4, Franz Faul, Universität Kiel, Germany). According to a previous study, a total sample size of 42 subjects is required to obtain 80% power with d = 0.3930510 effect size, α = 0.05 type I error, and β = 0.20 type II error [26].

## 3. Results

The study included 47 healthy individuals (24 males and 23 females) between the ages of 18 and 25 years. The mean ages of the males and females were 20.08 ± 1.44 years and 19.57 ± 0.90 years, respectively. There was no significant difference in age between males and females (p *>* 0.05). Eleven of the males (45.8%) were right-handed and 13 (54.2%) were left-handed, whereas 9 of the females (39.1%) were right-handed and 14 (60.9%) were left-handed. Handedness was statistically similar in males and females (p *>* 0.05) (Table 1).

**Table 1:** Demographic characteristics of participants.

Characteristics (n = 47)		Mean ± Std. deviation
Age (years)	F	19.57 ± 0.90
	M	20.08 ± 1.44
Sex (n, %)	F	23/48.9%
	M	24/51.1%
Handedness (n, %)	R	20/42.6%
	L	27/57.4%
Total brain (GM + WM) (cm3)		1315.57 ± 121.53
Total cerebrum (cm3)		1148.27 ± 106.79

GM: grey matter, WM: white matter.

The right insular volume was significantly higher than the left insular volume, and the left cerebral volume was significantly higher than the right cerebral volume (p *<* 0.05) (Table 2). The total brain, total cerebral, left and right insular, and left and right cerebral volumes were significantly larger in males than females (p *<* 0.001) (Table 3).

**Table 2:** Volume differences between sides of insula and cerebrum using ??

Characteristics (n = 47)		Mean	Std.deviation	Mean difference[95% CI]/z	t	p
Insula (cm3)	Left	7.82	1.71	–4.25		<0.001
	Right	8.25	1.35			
Cerebrum (cm3)	Left	575.10	53.46	0.59 [(0.72)–( 3.14)]	3.22	0.002
	Right	573.17	53.40			

**Table 3:** Sex differences of volumetric morphometry of total brain, total cerebrum, insula and cerebrum.

		Male (N:15)Mean ± SD	Female (N:15)Mean ± SD	Mean difference [95%CI]/u	p
Total brain (GM+WM)(cm3)		1386.91 ± 78.23	1244.21 ± 115.95	142.70[(83.92) –( 201.48)]	<0.001
Total cerebrum (cm3)		1209.72 ± 72.14	1086.81 ± 101.11	122.90[(70.70)–( 175.10)]	<0.001
Insula (cm3)	Left	8.68 ± 1.96	6.93 ± 0.68	52	<0.001
	Right	9.02 ± 1.46	7.46 ± 0.57	44	<0.001
Cerebrum (cm3)	Left	605.76 ± 36.77	544.44 ± 50.29	61.32[(35.14)–( 87.49)]	<0.001
	Right	603.96 ± 35.48	542.37 ± 50.91	61.58[(35.50)–( 87.66)]	<0.001

GM: grey matter, WM: white matter.

**Figure 6:**
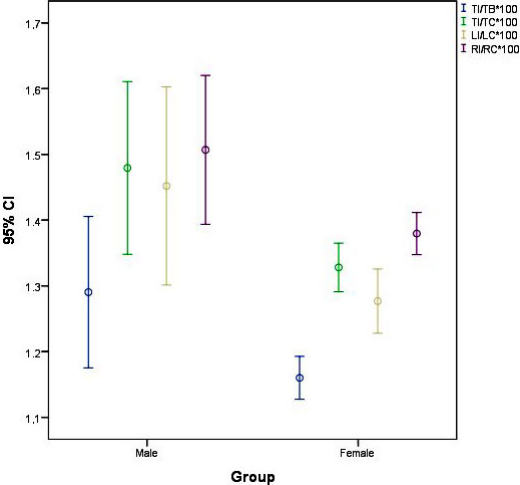
Comparisons of ratio of TI/TB, TI/TC, LI/LC, RI/RC between sexes. TI: total insular volume, TB: total brain volume, TC: total cerebral volume, LI: left insular volume, LC: left cerebral volume, RI: right insular volume, RC: right cerebral volume.

The ratio of the insular volume to total brain volume was 1.2904 ± 0.2663 in males and 1.1601 ± 0.0754 in females; insular volume to total cerebral volume was 1.4794 ± 0.3039 in males and 1.3281 ± 0.0854 in females; left insular volume to left cerebral volume was 1.4520 ± 0.3487 in males and 1.2768 ± 0.1131 in females; and, right insular volume to right cerebral volume was 1.5069 ± 0.2618 in males and 1.3796 ± 0.0742 in females. All of the ratio values were significantly higher in males than in females (p *<* 0.05) (Figure 6). 

## 4. Discussion

The main findings of the present study are as follows: (1) The total brain, total cerebral, total insular, and left and right insular and cerebral volumes were greater in male participants than in female participants. (2) The right insular volume was greater than the left insular volume, while the left cerebral volume was greater than the right cerebral volume in all participants. (3) The ratios of the insular volume to total brain volume and cerebral volume, left insular volume to left cerebral volume, and right insular volume to right cerebral volume were higher in male participants than in female participants. 

Several MRI studies on human cortical and subcortical development have revealed sex differences [15–17]. Understanding the effect of sex on brain development may help understand the sex differences seen in the development of psychopathological conditions. During brain development, biological and environmental factors result in sex differences in brain structure [27]. A meta-analysis showed that males have higher tissue densities in the insular cortex, while females have greater insular volumes. In addition, in males, bilateral limbic areas and left posterior cingulate gyrus volumes are mostly greater, while only the left side of the limbic system has a higher density. Conversely, the volumes of the right hemisphere (related to language), right insular cortex and anterior cingulate gyrus are greater in females [18]. Through MRI-based analysis, Kennedy et al. found that insular volume was 17.6 ± 2.1 cm3 in males and 16.9 ± 1.7 cm3 in females [28]. In another study, MRI-based analysis revealed a larger insular gyrus in males than in females. The authors stated that males may have a greater insular surface and/or volume as they have a larger and heavier brain. In addition, according to their study, besides the larger insula, the gyrus pattern is larger in males [29]. Similar to the study by Kennedy et al., in the present study, the insular volume was greater in males than in females. In addition, our study showed for the first time that the ratios of the insular volume to total brain volume and cerebral volume, left insular volume to left cerebral volume, and right insular volume to right cerebral volume were higher in males than in females. The participants in the present study and in the study by Kennedy et al. [28] were younger than the participants in the meta-analysis, who belonged to a wide age range [18]. 

The insula makes reciprocal connections with the limbic system [5]. The integration of perceptual experiences is a critical function of the insular cortex, which is a part of the limbic region. Therefore, the insular cortex is responsible for balanced behaviour [30]. The anterior insular lobule is primarily responsible for higher cognitive and emotional tasks, rather than simple motor activities, while the posterior insular lobule is sensitive to general somatosensory, temperature and pain stimuli; these lobules show a distinct somatotopic organisation [3,7,31,32]. The anterior and posterior insular volumes may be different in males and females. Therefore, studies investigating the sex differences in anterior and posterior insular volumes need to be conducted. We could not measure the anterior and posterior insular volumes due to limitations of the system we used (IBASPM). Systems that measure the anterior and posterior insular volumes separately need to be developed. 

There is a functional difference between the left and right anterior insula. The right anterior insula is associated with negative emotional valence and sympathetic activation, while the left anterior insula is associated with positive emotional valence and parasympathetic function [1,8]. In a previous study, it was stated that the left insular volume was 8.8 ± 0.9 cm3 and the right insular volume was 8.5 ± 1.1 cm3 in young adult participants [28]. In another study, the mean number of right insular gyri was found to be 4.39, while that of left insular gyri was found to be 4.46 [29]. In contrast, in the present study, the right insular volume (7.95 ± 0.82 cm3) was found to be greater than the left insular volume (7.43 ± 0.96 cm3). This finding may be explained by the relationship between behavioural functions and emotional reactions and the insular anatomy, and by the fact that the right brain is responsible for expressive and creative tasks [33]. 

Diverse software tools, such as FMRIB Software Library (FSL) [34], FreeSurfer [35], Statistical Parametric Mapping (SPM) [36], MRIcroGL [1], Individual Brain Atlases toolbox (IBASPM; Cuban Neuroscience Center) [37], and volBrain [38] are available for volumetric measurements of brain structures. IBASPM, which was used in the present study, is a web-based software for the automatic segmentation of individual MRI images into different anatomical structures using a standardised atlas. Two studies comparing IBASPM and FreeSurfer indicated that the average errors in hippocampal measurements taken using IBASPM were lower, but more widely distributed than those in hippocampal measurements taken using FreeSurfer [23,39]. The other program we used was volBrain, an online MRI brain volumetry system that automatically obtains volumetric brain information from researchers’ 3D MRI data, without the need for any infrastructure at their local sites. Moreover, volBrain is fully automatic and provides volumes of the specific brain parts without any human interaction [20]. 

Several changes in the cortical and subcortical morphometry have been reported in different diseases using segmentation techniques. The insular cortex plays an important role in the development of schizophrenia, mood disorders, eating disorders, obsessive-compulsive disorder and panic disorders. The insular volume of the individuals with psychiatric disorders was found to be lower than that of the healthy individuals [1,32,33,40,41]. Knowledge of the normal insular volume and how this differs with sex and lateralisation could aid the early diagnosis of such disorders. Moreover, such information might be important in clinical examinations to help determine the causes of symptoms and surgical success. Studies of anatomical dissections, and structural and functional imaging analysis have led to a reduction in perioperative morbidity in insular surgery [42]. Together with multimodal imaging technology to map brain function, this has widened surgical possibilities enabling transcortical resection of insular tumours [43]. 

In conclusion, to our best knowledge, this is the first study that compares the ratio of insular volume to total brain and cerebral volume. Moreover, we are the first to show sex- and laterality-based differences in insular volume. However, the present study has some potential limitations. First, the anterior and posterior insula and grey/white matter volumes could not be measured using IBASPM. Different automatic segmentation programs will need to be used for detailed analysis of the insular volume. Second, the present study included younger individuals only; therefore, the results cannot be generalised. In future studies, sex differences should be investigated in different age groups. In addition, our findings need to be confirmed in a larger population. 

## Informed consent

The study was approved by the local ethics committee (Akdeniz University, protocol no.: 2015.02.25) and written informed consent was obtained from each subject, in accordance with the Declaration of Helsinki.
